# Transgenic Canola Oil Improved Blood Omega-3 Profiles: A Randomized, Placebo-Controlled Trial in Healthy Adults

**DOI:** 10.3389/fnut.2022.847114

**Published:** 2022-03-10

**Authors:** Xinjie Lois Lin, Joshua Baisley, Anthony Bier, Drasty Vora, Bruce Holub

**Affiliations:** ^1^Nutrasource Pharmaceutical and Nutraceutical Services Inc., Guelph, ON, Canada; ^2^Department of Human Health and Nutritional Sciences, University of Guelph, Guelph, ON, Canada

**Keywords:** omega-3, transgenic canola oil, docosahexaenoic acid (DHA), eicosapentaenoic acid (EPA), pharmacokinetics, bioavailability, lipid profile, cardiovascular biomarkers

## Abstract

Long-chain omega-3 polyunsaturated fatty acids (LC-ω3 PUFA), including eicosapentaenoic acid (EPA) and docosahexaenoic acid (DHA), play key roles in physiological functions and disease prevention. The nutrient gap in meeting LC-ω3 intake recommendations in the U.S. and globally can be addressed by alternative sources of LC-ω3. This randomized, placebo-controlled, seamless phase I/II study evaluated the pharmacokinetics, safety, and efficacy of a transgenic LC-ω3-rich canola oil in healthy adults. Participants (*n* = 33/group) were randomized to receive low-, mid-, or high-dose of the LC-ω3-rich oil (providing 285, 570, or 1,140 mg LC-ω3 PUFA, respectively) or placebo (corn oil). After one dose, plasma ω3 (primary outcome) levels were assessed over a 72 h pharmacokinetic period. Whole blood and red blood cells (RBC) ω3 and serum cardiovascular biomarkers were assessed during a 16-week continuation period with daily supplementation. Compared to low-dose and placebo, high-dose group showed greater DHA AUC_0−72*h*_ and C_*max*_. A linear response was observed for DHA and EPA AUC_0−72*h*_. Compared to placebo, high- and mid-dose groups showed increased whole blood DHA, EPA, α-linolenic acids (ALA) (high-dose only), omega-3 score, and omega-3 index after 4 weeks, and increased DHA and EPA in RBC after 16 weeks (*P* < 0.05). No changes in cardiovascular biomarkers were seen. Overall, this LC-ω3-rich oil demonstrated good DHA bioavailability and significantly improved short and long-term blood LC-ω3 profiles. Sixteen weeks of daily supplementation of the LC-ω3-rich oil was safe and well-tolerated.

## 1. Introduction

Fats and fatty acids are considered key nutrients affecting both early growth and development, as well as nutrition-related chronic diseases later in life. Indeed, the blood lipid and fatty acid profiles play critical roles in human health and they can be modulated by dietary changes, such as increasing long-chain omega-3 polyunsaturated fatty acid (LC-ω3 PUFA) intake (1). The physiological functions, and subsequent health benefits, of LC-ω3 PUFA result from numerous biological mechanisms through their integral roles in cell membrane composition, regulation of gene expression and serving as precursors to hormones that regulate blood clotting, contraction and relaxation of artery walls, cell division and growth and inflammation (2). Furthermore, a recent study showed that LC-ω3 PUFA supplementation altered gut microbiome composition and gut microbial fermentation products in healthy adults, indicating another potential mechanistic pathway for these fatty acids (3). Overall, LC-ω3 PUFA play a key role in supporting health and preventing disease (2).

There is a large nutrient gap between the current dietary intake, ~80–110 mg/person/day, of LC-ω3 PUFA [i.e., docosahexaenoic acid (DHA) and eicosapentaenoic acid (EPA)] in the U.S. population (4–7) and the in take guidelines of 250–500 mg/day for healthy adults (8, 9). Increased awareness of the role LC-ω3 PUFA play in human health and disease risk drives a growing interest. The global demand for LC-ω3 PUFA exceeds 1.27 million tones per year, based on the most commonly recommended dose for cardiovascular health (500 mg/person/day) by the Global Organization for EPA and DHA Omega 3s (GOED) (10). The supply is estimated at 0.2 (11) to 0.8 (12) million tones per year from the ocean highlighting a shortage of more than 0.4–1 million tones (12).

DHA and EPA are found in marine sources like fatty fish (e.g., salmon, smelt, and anchovies), and fish oil capsules; however, these sources tend to be under-consumed due to unpalatability, accessibility issues, public concerns with respect to environmental pollutants (e.g., mercury, polychlorinated biphenyls, chlordane, dioxins, etc.) as well as vegetarian or vegan dietary preferences (13). Alternative sources of DHA and EPA are necessary for more than consumer preferences; the finite supply of ocean fish is not sufficiently sustainable or scalable to meet global requirements as described above. Fermented and genetically modified (GM) plant-based sources of LC-ω3 PUFA are being developed (e.g., algae and oilseeds), with land-based oilseed products having the advantage of a well-established infrastructure (12). A land-based LC-ω3 PUFA-enriched oil from GM Camelina sativa has shown safety and bioavailability in humans (14), demonstrating the potential to have more land-based LC-ω3 PUFA sources that are widely available and consumed. Canola oil, an already popular cooking oil, has a favorable fatty acid profile (low saturated fatty acids) and is a good source of α-linolenic acid (ALA), an essential ω3 fatty acid that can be converted to EPA and DHA in humans. However, the conversion efficiency is limited, making ALA a poor precursor for the subsequent production of EPA and even more so for DHA (15). A transgenic canola oil with additional LC-ω3 PUFA will be an easily scalable and accessible source without adding pressure to marine resources. This study was designed to investigate the bioavailability of LC-ω3 PUFAs from a new canola oil source to evaluate its role in contributing to health benefits attributed to other ω3 fatty acid sources.

Described here is a two-phase study that evaluated the acute bioavailability of DHA and EPA from a transgenic, LC-ω3-rich canola oil within 72 h of consuming a single dose (the phase I period) followed by a daily supplementation for 16 weeks (the phase II period). ω3 profiles, which are measurements of ω3 bioavailability, were assessed in plasma and whole blood after 4 weeks, and in red blood cells (RBC) after 16 weeks (to allow for complete RBC turnover).

## 2. Materials and Methods

### 2.1. Participants

The study was approved by the Canadian Shield Ethics Review Board (OHRP Registration IORG0003491, Burlington, Ontario) and Health Canada's Natural and Non-Prescription Health Products Directorate (Ottawa, Ontario), and registered at ClinicalTrials.gov (NCT03937206). The study was conducted at one Canadian clinical research site in Guelph, Ontario. All participants gave written informed consent and were free to withdraw from the study at any time without consequence.

Two hundred and twenty individuals were screened for eligibility. The key inclusion criteria for the study were: healthy adults, aged 18–80 years of age (inclusive), body mass index (BMI) of 18.5–34.9 kg/m^2^ at the time of screening, and consumption of <200 mg ω3 per day based on a validated questionnaire (15). Individuals were excluded from the study if they had consumed fish 2 weeks prior to the first dose of study product or used canola oil, fish oil, or other ω3 fatty acid (DHA and/or EPA) containing supplements within 1 month of baseline and refused to refrain from consuming such during the study period. Also excluded were individuals with a history or presence of any condition known to interfere with absorption, distribution, metabolism, or excretion of drugs, as well as taking prescription or non-prescription health products such as corticosteroids, anti-inflammatory drugs, and blood lipid-lowering drugs (e.g., statins, fibrates, bile acid exchanger resin, phytosterols, niacin or its analogs, carnitine, etc.) within 6 months prior to the screening visit.

### 2.2. Study Design and Study Products

This was a prospective, randomized, double-blind, 4-arm parallel-group study in healthy adults to evaluate the test product (TP), a LC-ω3-rich canola oil (Nutriterra^Ⓡ^ Total Omega-3 from transgenic canola seeds, developed by Nuseed^Ⓡ^). This study included two phases; participants started with phase I (one-time dosing) for pharmacokinetics (PK) assessments and then entered phase II (daily dosing for 16 weeks) for efficacy and safety assessments. Three different doses of the TP were compared to placebo. Randomization to the four study groups in a 1:1:1:1 ratio was performed using a computer-generated algorithm and randomization numbers were allocated to participants using a site-specific paper-based randomization log. The product identity was blinded to the study staff and the participants. The TP and placebo capsules were similar in size and shape and were filled in similar primary packaging to minimize bias.

Each TP capsule contained 1,000 mg of the LC-ω3-rich canola oil, providing at least 300 mg/g ω3 fatty acids [DHA, EPA, ALA, stearidonic acid, docosapentaenoic acid (DPA), eicosatetraenoic acid, and eicosatrienoic acid] in the form of triglycerides (TG) and ~1,500 ppm of mixed natural tocopherols (from soy) as a natural anti-oxidant. Each placebo capsule contained 1,000 mg of corn oil, in which the PUFA are mainly ω6 (linoleic acid, >60% total fatty acids) with only a trace amount of ω3 (ALA, <1.5% total fatty acids) (16). The inactive ingredients of all capsules included purified water, modified corn starch, glycerin (anhydrous), carrageenan, sodium phosphate di-basic, and mixed tocopherols. Details of study products consumed in each study group is provided in Table 1.

**Table 1:** Study products consumed in each study group.

	**High-dose**	**Mid-dose**	**Low-dose**	**Placebo**
TP capsules per dose (*n*)	4	2	1	0
Placebo capsules per dose (*n*)	0	2	3	4
DHA (mg)	360	180	90	–
EPA (mg)	20	10	5	–
ALA (mg)	760	380	190	Trace
DPA (mg)	40	20	10	–
Total ω3 (mg)	^≥^1200	^≥^600	^≥^300	Trace

*ALA, alpha-linolenic acid; DHA, docosahexaenoic acid; EPA, eicosapentaenoic acid; mg, milligram; n, number; ω3, omega-3; TP, test product (Nutriterra^Ⓡ^ Total Omega-3)*.

### 2.3. Study Procedures and Clinical Measurements

The study included a screening visit (visit 1), a PK baseline visit (visit 2, start of phase I), a 72 h PK period (visits 3, 4, and 5, visit 5 being the end of phase I), followed by a 2-week washout period and a 16-week efficacy phase (phase II, visits 6–10). The screening period was up to 28 days for eligibility assessment but extended to up to 90 days for menstruating women, in order to obtain their baseline measurements during the mid-cycle phase to reduce the potential variability in lipid metabolism and pharmacokinetics due to menstruation (17, 18). During visit 2, the participants attended an in-clinic visit after fasting overnight for at least 12 h. A standardized meal was provided 30 min before the study product administration. PK blood draws were performed prior to dosing and at pre-specified timepoints after a single dose of study product (four capsules) was administered (1.0, 2.0, 3.0, 4.0, 5.0, 6.0, 7.0, 8.0, 10.0, 12.0 h). Participants returned to the clinic for post-dose PK blood draws at 24±1 (visit 3), 48±1 (visit 4), and 72±1 (visit 5) hours after dosing and after at least 12 h of fasting before sampling. The primary PK endpoint was plasma ω3 profiles (DHA, EPA, ALA, and DPA).

After a minimum of 2-week washout period, the participants entered the efficacy phase, in which they started daily supplementation of assigned study products at visit 6 (baseline of the efficacy phase) and ended at visit 10. During the efficacy phase, fasting blood samples (≥12 h) were collected on visits 7 and 10 for the assessment of primary and secondary efficacy endpoints. Specifically, whole blood ω3 profiles were measured at visit 7 (4 weeks after baseline), RBC ω3 profiles were measured at visit 10 (16 weeks after baseline), and lipid profile [including TG (primary efficacy endpoint), total cholesterol (TC), low-density lipoprotein-cholesterol (LDL-C), high-density lipoprotein-cholesterol (HDL-C), non-HDL-C, and LDL-C/HDL-C ratio] and high sensitivity C-reactive protein (hsCRP) were measured at both visits 7 and 10. A phone follow-up was performed 14 ± 3 days after visit 10 to follow up on any adverse events. Study product compliance was maintained by dosing the participants at the site during the visits as per the schedule, and by providing a diary for participants to record the daily study product use at home. Unused study products were collected, reviewed and compliance was calculated by returned capsule count during every study visit. Noncompliance was defined as an overall study product compliance <80% throughout the study, use of prohibited drugs or other products that affect the primary efficacy endpoint during the study, and/or early discontinuation of study or study product before visit 10.

All blood sample analyses were done at commercial bioanalytical laboratories. For lipid profile and hsCRP, blood was collected in serum separating tubes and stored at 2–8°C before analysis by Cobas C701 (Roche, Switzerland) at Lifelabs medical laboratory services (Ontario, Canada). For plasma, whole blood, and RBC fatty acid analyses, blood was collected with K2 EDTA anticoagulant, and RBC samples were separated by centrifugation at 3,200 rpm for 10 min. Plasma and RBC samples were stored at −80°C. Plasma ω3 fatty acids were assessed using a validated method developed by Altasciences (Quebec, Canada). Specifically, stable-isotope labeled internal standards were added to diluted plasma samples under acid and base conditions for the ω3 free fatty acids and deconjugation of ω3 TG; EPA and DHA were assessed by high performance liquid chromatography on an XBridge C18 column (50 × 2.1 mm, 5 μm) with a mobile phase composition of acetic acid and methanol, and DPA and ALA by ultra-performance liquid chromatography (UPLC) on an Acquity UPLC C18 column (100 × 2.1 mm, 1.7 μm) with a mobile phase of acetic acid and acetonitrile. Parent ions were formed by negative ion spray mode for all analytes and its internal standards with the multiple reaction monitoring transitions mass/charge ratios: 301.2 → 257.2 (EPA), 327.2 → 283.2 (DHA), 306.2 → 262.2 (EPA-d5), 332.2 → 288.2 (DHA-d5), 329.2 → 231.2 (DPA), 277.2 → 233.2 (ALA), 334.2 → 236.2 (DPA-d5), and 282.2 → 238.2 (ALA-d5), using a SCIEX API5500 system. The theoretical concentration ranges of detection for EPA, DHA, DPA, and ALA were 2.00–200, 10.0–200, 5.00–100, and 5.00–200 μg/mL, respectively. For whole blood, analyses of fatty acid composition were performed at a central laboratory (University Health Network, Specialty Laboratory, Toronto, Ontario, Canada). Specifically, lipids were extracted from 50 μL of sample and then transesterified by a 3 h incubation with 1% sulfuric acid in methanol solution at 70°C (19). After cooling, fatty acid methyl esters were extracted with heptane. The fatty acid composition was then determined by gas chromatography performed on a 100-m Varian SelectTM FAME CP7420 capillary column (0.25 mm internal diameter), using an Agilent Technologies 6890N series gas chromatograph equipped with a split/splitless mode injector (maintained at 280°C) and a flame ionization detector (300°C). Samples were analyzed by multilevel temperature programming in the range of 100–285°C with ultra-high purity grade helium as the carrier gas. The percent composition of fatty acids was calculated from the individual peak areas using appropriate reference standards. Total omega-3 was defined as ALA, eicosatrienoic acid, EPA, DPA n-3, and DHA, and total omega-6 as DPA n-6, adrenic acid, docosadienoic acid, arachidonic acid, dihomo-gamma-linolenic acid, eicosadienoic acid, and linoleic acid; omega-3 to omega-6 ratio was calculated. Whole blood omega-3 score was calculated by summing the percent composition of whole blood EPA, DPA n-3, and DHA. Omega-3 index was calculated *via* a correlation equation from the sum percent composition of whole blood DHA+EPA (20):


$$\begin{array}{l} \text{Omega-3}\;\text{index}=\text{whole blood DHA + EPA }(\%)\\ \;\times \text{ 0}.\text{9096 + 2}.\text{1316} \end{array}$$

RBC fatty acid profile was analyzed with a verified method developed by Diteba Laboratories Inc. (Ontario, Canada). Lipids in the RBC samples were derivatized by boron trifluoride-methanol (21), extracted by heptane, and analyzed by gas chromatograph [Varian 3900 with a DB-Wax column (30 m × 0.25 mm ID, 0.15 μm film)] equipped with a split/splitless mode injector (250°C) and a flame ionization detector (300°C). Multilevel temperature programming in the range of 170–245°C was applied with helium as the carrier gas. Fatty acids were identified using an external standard (GLC-566B, Nu-Chek Prep, Minnesota, USA).

### 2.4. Power Analysis and Statistical Methods

A minimum of 33 participants for each study group was sufficient for assessing the PK and safety of the TP, while accounting for participant withdrawal (22). An interim analysis was done by a blinded biostatistician on whole blood ω3 profile (DHA, EPA, DPA, and ALA) after visit 7 (4 weeks) to confirm the TP modulated ω3 profiles as expected. Data analysis was performed using SAS version 9.4.

For the phase I data, baseline-corrected PK parameters [AUC_0−72*h*_, maximum concentration (C_*max*_), log[AUC_0−72*h*_], log[C_*max*_], and time at which the C_*max*_ is observed (T_*max*_) for plasma DHA and EPA; AUC_0−72*h*_, C_*max*_, and T_*max*_ for plasma DPA and ALA] were analyzed and presented. T-tests were applied to baseline-corrected PK parameters (AUC_0−72*h*_ and C_*max*_) to compare between each dose of TP and placebo. To examine differences between different doses of TP, the same *t*-test approach was applied to log-transformed and baseline-corrected AUC_0−72*h*_ and C_*max*_. Mann–Whitney *U*-tests were performed to compare T_*max*_ between groups (23). To determine whether the effects of TP increased proportionally with doses, analysis of variance (ANOVA) (24) was used to compare the log-transformed and dose-adjusted means of AUC_0−72*h*_ and C_*max*_ of DHA and EPA in the TP groups. All doses of TP were adjusted to high-dose as following: high-dose: dose adjusted AUC_0−72*h*_ (or C_*max*_) = 1 × AUC_0−72*h*_ (or C_*max*_); mid dose: dose adjusted AUC_0−72*h*_ (or C_*max*_) = 2 × AUC_0−72*h*_ (or C_*max*_); low dose: dose adjusted AUC_0−72*h*_ (or C_*max*_) = 4 × AUC_0−72*h*_ (or C_*max*_). Linear regressions were also performed with log[AUC_0−72*h*_] and log[C_*max*_] as dependent variables with log(dose) as the independent variable in order to explore any linear relationship between doses and PK parameters.

For the phase II data, efficacy parameters (e.g., omega-3 score, TG, LDL-C, etc.) were analyzed using analysis of co-variance (ANCOVA) (25) with their change from baseline at different visits as the responsible variable, treatment, visit, and smoking status as fixed effects, and the baseline value as a covariate. *P* < 0.05 was considered statistically significant for all analyses. Data are reported as mean ± standard deviation (SD).

## 3. Results

### 3.1. Participants

**Figure 1:**
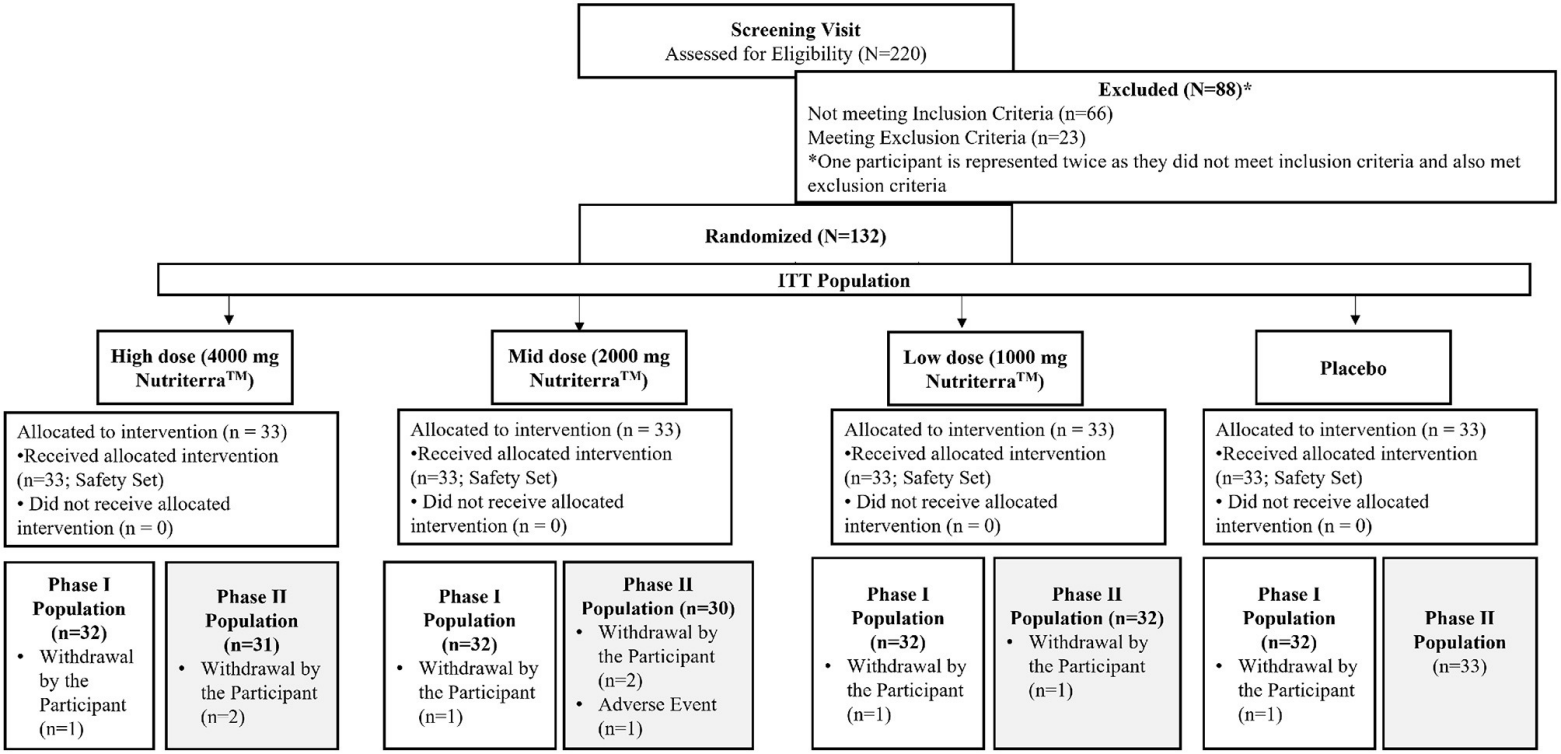
CONSORT diagram for participant disposition.

Figure 1 presents the Consolidated Standards of Reporting Trials (CONSORT) flow diagram of the study (26), which was initiated on 01-May-2019 and was completed on 27-December-2019. A total of 132 participants who passed the screening were randomized in a 1:1:1:1 ratio into the four study groups, each containing 33 participants who received one of the three different doses of TP or placebo. Participant characteristics at screening are summarized in Table 2. Five participants were removed from the study due to non-compliance; the remaining participants were compliant to the study procedures. The mean study product compliance across all study groups for the whole study was 95.7%, and compliance was generally comparable across study product groups and study visits. Given that the study participants went through both phase I and phase II of the study, two study populations were defined for the analyses of phase I (PK) and phase II (efficacy) study endpoints. The phase I population was consisted of all participants who completed the PK test visit and had no major protocol deviations during the period that would have invalidated PK data, and the phase II population included all participants who received at least one dose of study product and had at least one post-randomization efficacy assessment.

**Table 2:** Characteristics of enrolled participants at screening.

	**High-dose** **(*n* = 33)**	**Mid-dose** **(*n* = 33)**	**Low-dose** **(*n* = 33)**	**Placebo** **(*n* = 33)**	**Total** **(*n* = 132)**
Gender (%)					
Women	19 (57.6%)	26 (78.8%)	22 (66.7%)	25 (75.8%)	92 (69.7%)
Men	13 (39.4%)	7 (21.2%)	11 (33.3%)	8 (24.2%)	39 (29.5%)
Other	1 (3.0%)	0 (0.0%)	0 (0.0%)	0 (0.0%)	1 (0.8%)
Age (Year)	37.8 ± 15.7	36.6 ± 14.1	33.9 ± 13.2	34.2 ± 12.2	35.7 ± 13.8
Height (cm)	169.8 ± 9.1	165.8 ± 7.3	169.9 ± 9.9	166.9 ± 8.3	168.1 ± 8.8
Weight (kg)	74.2 ± 11.3	68.6 ± 12.0	73.2 ± 15.1	71.7 ± 12.9	71.9 ± 12.9
Body mass index (kg/m^2^)	25.7 ± 3.3	24.8 ± 3.1	25.2 ± 3.8	25.6 ± 3.2	25.3 ± 3.3
Diastolic blood pressure (mmHg)	78.0 ± 8.8	73.0 ± 7.6	75.2 ± 6.7	73.6 ± 8.8	74.9 ± 8.1
Systolic blood pressure (mmHg)	122.6 ± 11.7	116.7 ± 10.1	122.5 ± 10.2	118.2 ± 10.6	120.0 ± 10.9

*Values are means ± SD. n, number of participants in group*.

### 3.2. High-Dose LC-ω3-Rich Canola Oil Supplementation Increased DHA and ALA Bioavailability Over 72 h Post-dose

**Figure 2:**
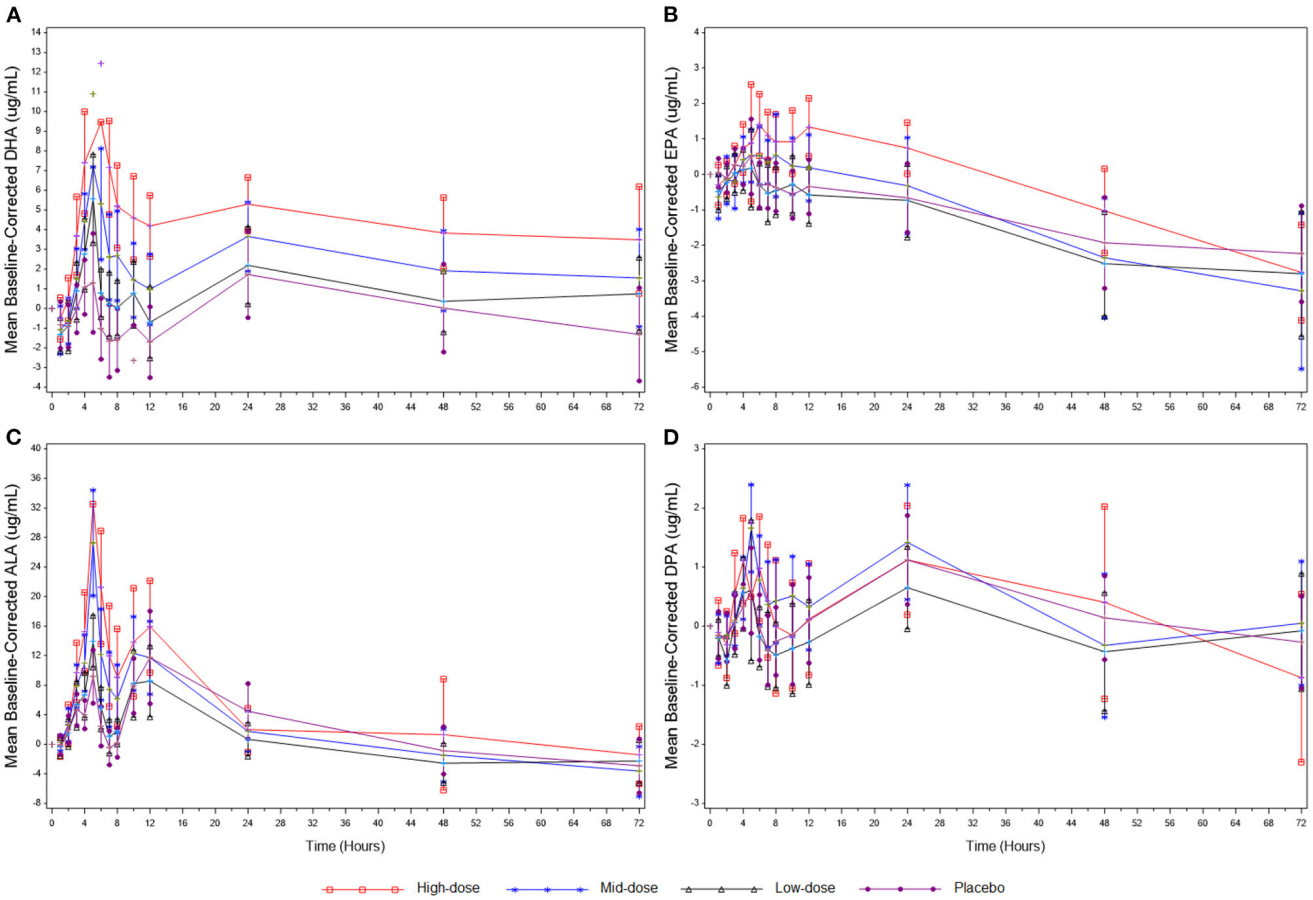
**(A)** Mean baseline-corrected plasma concentrations of DHA during the 3-day PK phase. **(B)** Mean baseline-corrected plasma concentrations of EPA during the 3-day PK phase. **(C)** Mean baseline-corrected plasma concentrations of ALA during the 3-day PK phase. **(D)** Mean baseline-corrected plasma concentrations of DPA during the 3-day PK phase. Values are means ± SD. ALA, alpha-linolenic acid; DHA, docosahexaenoic acid; DPA, docosapentaenoic acid; EPA, eicosapentaenoic acid; PK, pharmacokinetics.

The PK baseline-corrected mean concentrations for DHA, EPA, ALA, and DPA are presented in Figure 2 and Table 3. For DHA, mean AUC_0−72*h*_ was greater in the high-dose group compared to the low-dose (*P* = 0.0003) and placebo (*P* = 0.0032) groups, and mean C_*max*_ was higher in the high-dose group compared to the mid-dose (*P* = 0.0002), low-dose (*P* < 0.0001), and placebo (*P* < 0.0001) groups, as well as in the mid-dose group compared to the low-dose (*P* = 0.0218) and placebo (*P* = 0.0003) groups over a 72 h period. No significant difference between groups in AUC_0−72*h*_ or C_*max*_ were found for EPA, and no significant differences between groups in T_*max*_ were found in either DHA or EPA.

**Table 3:** Baseline-corrected plasma PK parameters for DHA, EPA, ALA, and DPA within 72 h post-dose of LC-ω3-rich canola oil.

		**High-dose**	**Mid-dose**	**Low-dose**	**Placebo**
DHA	AUC_0−72*h*_ (μg*h/mL)	360.60 ± 211.63^a^	269.86 ± 233.19^ab^	175.57 ± 165.49^b^	193.49 ± 207.30^b^
	C_*max*_ (μg/mL)	21.18 ± 9.51^a^	13.14 ± 6.44^b^	9.40 ± 5.96^c^	6.72 ± 6.23^c^
	T_*max*_ (hour)	11.38 ± 17.89^a^	8.71 ± 12.68^a^	15.97 ± 23.12^a^	17.82 ± 16.50^a^
EPA	AUC_0−72*h*_ (μg*h/mL)	78.21 ± 74.62^a^	70.50 ± 87.12^a^	57.52 ± 85.91^a^	52.68 ± 87.00^a^
	C_*max*_ (μg/mL)	3.47 ± 2.55^a^	3.18 ± 2.93^a^	3.05 ± 2.77^a^	2.87 ± 3.09^a^
	T_*max*_ (hour)	14.93 ± 17.94^a^	15.00 ± 18.65^a^	14.08 ± 19.98^a^	19.00 ± 21.04^a^
ALA	AUC_0−72*h*_ (μg*h/mL)	533.07 ± 560.08^a^	362.86 ± 279.39^ab^	249.61 ± 237.92^b^	346.89 ± 379.87^ab^
	C_*max*_ (μg/mL)	45.55 ± 25.53^a^	33.51 ± 16.84^b^	19.82 ± 12.08^c^	19.70 ± 17.98^c^
	T_*max*_ (hour)	8.06 ± 10.60^a^	8.35 ± 12.07^a^	8.38 ± 7.87^a^	11.42 ± 8.70^a^
DPA	AUC_0−72*h*_ (μg*h/mL)	107.25 ± 126.82^a^	82.48 ± 94.25^a^	67.40 ± 69.29^a^	75.70 ± 82.54^a^
	C_*max*_ (μg/mL)	4.28 ± 3.39^a^	3.33 ± 2.65^a^	2.80 ± 2.52^a^	2.81 ± 2.33^a^
	T_*max*_ (hour)	18.87 ± 22.57^a^	19.67 ± 22.35^a^	25.34 ± 24.77^a^	23.45 ± 21.49^a^

*Values are means ± SD. For each parameter, different letters (a,b,c,d) indicates statistically significant differences between groups (P < 0.05); t-test was applied to baseline-corrected AUC_0−72h_ and C_max_, and Mann–Whitney U-test was applied to T_max_. For DHA, n = 32, 31, 30, and 28 for high-dose, mid-dose, low-dose, and placebo groups; for EPA, n = 30, 28, 26, and 26 for high-dose, mid-dose, low-dose, and placebo groups; for ALA, n = 32, 31, 32, and 31 for high-dose, mid-dose, low-dose, and placebo groups; for DPA, n = 31, 30, 29, and 29 for high-dose, mid-dose, low-dose, and placebo groups. ALA, alpha-linolenic acid; AUC, area under the curve; C_max_, peak concentration; DHA, docosahexaenoic acid; DPA, docosapentaenoic acid; EPA, eicosapentaenoic acid; PK, pharmacokinetics; T_max_, time at which the C_max_ is observed*.

For ALA, mean AUC_0−72*h*_ was significantly greater in the high-dose group compared to the low-dose group (*P* = 0.0117), and mean C_*max*_ was higher in the high-dose group compared to the mid-dose (*P* = 0.0309), low-dose (*P* < 0.0001), and placebo (*P* < 0.0001) groups, as well as in the mid-dose group compared to the low-dose (*P* = 0.0004) and placebo (*P* = 0.0028) groups over a 72 h period. No significant between-group differences in AUC_0−72*h*_ or C_*max*_ were found for DPA, and no significant between-group differences in T_*max*_ were found in either ALA or DPA.

### 3.3. DHA Bioavailability Increased Linearly With Higher Doses of LC-ω3-Rich Canola Oil Supplementation After 72 h Post-dose

Regression analysis performed using log transformed PK parameters (log[C_*max*_] and log[AUC_0−72*h*_]) as dependent variables with log(dose) as the independent variable showed a significant result for DHA-AUC_0−72*h*_ (*P* = 0.0011), DHA-C_*max*_ (*P* < 0.0001), and EPA-AUC_0−72*h*_ (*P* = 0.0092), suggesting a linear relationship between the log(dose) and the above-mentioned logarithmic PK parameters. A linear relationship between the log(dose) and log[C_*max*_] was not observed for EPA-C_*max*_ (Supplementary Table 1). In addition, the log[AUC_0−72*h*_] of DHA and EPA showed dose proportionality across the three doses of LC-ω3-rich canola oil supplementation (DHA: *P* = 0.4302; EPA: *P* = 0.9947; Supplementary Table 2).

### 3.4. Four Weeks of LC-ω3-Rich Canola Oil Supplementation Improved the Whole Blood ω3 Profile

The whole blood ω3 profile after 4 weeks of LC-ω3-rich canola oil supplementation is presented in Table 4. The percent composition of DHA increased in all canola oil-supplemented groups (all *P* < 0.05), while no change was observed in the placebo group (*P* > 0.05). A between-group comparison revealed a dose-related response of the changes in DHA: high-dose > mid-dose > low-dose > placebo (*P* < 0.01 between any two groups). Within-group increases were also observed in the percent composition of EPA in high- and mid-dose groups (both *P* < 0.05), while the increase in the low-dose group and the decrease in placebo group did not reach statistical significance (*P* > 0.05). Compared between groups, a dose-related response was observed for the changes in EPA: high-dose = mid-dose > low-dose > placebo (*P* < 0.05 between any two groups except between high- and mid-dose groups). Regarding the percent composition of ALA, only the high-dose group showed an increase (*P* = 0.0327), which was different (*P* < 0.05) from the low-dose and placebo groups. No within- or between-group differences were observed for the change in DPA percent composition. The omega-3 to omega-6 ratio improved only in the high- and mid-dose groups (both *P* < 0.0001) and these improvements were different from the changes in low-dose and placebo groups (*P* ≤ 0.001). Both omega-3 whole blood score and omega-3 index increased in high- and mid-dose groups (both *P* < 0.0001) but not in the other groups, and a dose-related response was observed for the changes in both endpoints: high-dose > mid-dose > low-dose > placebo (*P* < 0.05 between any two groups).

**Table 4:** Whole blood ω3 profile before and after the 4-week supplementation of LC-ω3-rich canola oil or placebo.

	**Study visit**	**High-dose**	**Mid-dose**	**Low-dose**	**Placebo**
DHA (%)	Baseline	2.68 ± 0.67	2.68 ± 0.60	2.55 ± 0.57	2.85 ± 0.62
	Week 4	3.57 ± 0.70	3.18 ± 0.59	2.76 ± 0.54	2.80 ± 0.61
	Δ	0.89 ± 0.43^a^*^^	0.50 ± 0.35^b^*^^	0.21 ± 0.26^c^*^^	−0.06 ± 0.25^d^
EPA (%)	Baseline	0.65 ± 0.24	0.69 ± 0.38	0.64 ± 0.20	0.60 ± 0.16
	Week 4	0.83 ± 0.21	0.78 ± 0.29	0.66 ± 0.22	0.55 ± 0.18
	Δ	0.18 ± 0.19^a^*^^	0.09 ± 0.28^a^*^^	0.02 ± 0.16^b^	−0.05 ± 0.15^c^
ALA (%)	Baseline	0.61 ± 0.23	0.60 ± 0.23	0.61 ± 0.17	0.62 ± 0.24
	Week 4	0.70 ± 0.23	0.65 ± 0.21	0.60 ± 0.16	0.63 ± 0.18
	Δ	0.08 ± 0.19^a^*^^	0.05 ± 0.16^a^	−0.01 ± 0.16^a^	0.00 ± 0.21^a^
DPA (%)	Baseline	1.36 ± 0.23	1.26 ± 0.26	1.36 ± 0.25	1.28 ± 0.22
	Week 4	1.30 ± 0.19	1.24 ± 0.21	1.33 ± 0.26	1.28 ± 0.22
	Δ	−0.06 ± 0.09^a^*^^	−0.02 ± 0.13^a^	−0.02 ± 0.09^a^	−0.01 ± 0.08^a^
Omega-6 to Omega-3 ratio	Baseline	7.46 ± 1.05	7.62 ± 1.27	7.61 ± 1.16	7.34 ± 0.93
	Week 4	6.04 ± 0.67	6.71 ± 0.96	7.29 ± 0.83	7.54 ± 1.00
	Δ	−1.41 ± 0.91^a^*^^	−0.91 ± 0.86^b^*^^	−0.32 ± 0.80^c^*^^	0.10 ± 0.62^d^
Omega-3 whole blood score	Baseline	4.69 ± 0.79	4.63 ± 0.90	4.55 ± 0.67	4.73 ± 0.73
	Week 4	5.70 ± 0.78	5.20 ± 0.82	4.75 ± 0.66	4.62 ± 0.74
	Δ	1.01 ± 0.52^a^*^^	0.57 ± 0.59^b^*^^	0.20 ± 0.36^c^	−0.12 ± 0.38^d^
Omega-3 index	Baseline	5.16 ± 0.70	5.19 ± 0.72	5.04 ± 0.55	5.27 ± 0.62
	Week 4	6.13 ± 0.70	5.73 ± 0.67	5.24 ± 0.54	5.17 ± 0.61
	Δ	0.98 ± 0.45^a^*^^	0.54 ± 0.48^b^*^^	0.20 ± 0.27^c^	−0.10 ± 0.29^d^

*Values are means ± SD. Whole blood ω3 profile parameter changes were analyzed using ANCOVA with treatment and smoking status as fixed effects, and the baseline parameter value as a covariate. P_baseline_ was <0.01 and P_smoking_ was <0.05 for all parameters. Values without a common letter (a, b, c, d) indicate significant between-group differences (P < 0.05).*

* *Indicates significant within-group change from baseline to week 4 (P < 0.05). At baseline and week 4, n = 31, 30, 31, and 33 for high-dose, middose, low-dose, and placebo groups, respectively. ALA, alpha-linolenic acid; DHA, docosahexaenoic acid; DPA, docosapentaenoic acid; EPA, eicosapentaenoic acid; Δ, change from baseline*.

### 3.5. Sixteen Weeks of LC-ω3-Rich Canola Oil Supplementation Improved the Red Blood Cell ω3 Profile

The RBC ω3 profile after 16 weeks of LC-ω3-rich canola oil supplementation is presented in Table 5. The percent composition of DHA increased in high- and mid-dose groups (both *P* < 0.001), with a reduction in the placebo group (*P* = 0.0396) and no change in low-dose group (*P* > 0.05). A between-group comparison revealed a dose-related response for the changes in DHA: high-dose > mid-dose > low-dose > placebo (*P* < 0.05 between any two groups). A within-group increase was also observed in the percent composition of EPA in the high-dose group (*P* = 0.0039), with a reduction in the placebo group (*P* = 0.0341) and no changes in the mid- and low-dose group (*P* > 0.05). The between-group differences in EPA were as follows: high-dose = mid-dose > low-dose = placebo (*P* < 0.05 for high-dose vs. low-dose, high-dose vs. placebo, mid-dose vs. low-dose, and mid-dose vs. placebo). Regarding the percent composition of ALA, only the mid-dose group showed an increase (*P* = 0.0354), but it was not different (*P* > 0.05) from the other groups. All groups had a reduction in the percent composition of DPA (*P* < 0.0001), with a greater reduction in high- and mid-dose groups than those in low-dose and placebo groups (*P* < 0.05).

**Table 5:** Red blood cell ω3 profile before and after the 16-week supplementation of LC-ω3-rich canola oil or placebo.

	**Study visit**	**High-dose**	**Mid-dose**	**Low-dose**	**Placebo**
DHA (%)	Baseline	3.71 ± 0.83	3.83 ± 0.99	3.67 ± 0.92	4.03 ± 0.87
	Week 16	5.42 ± 1.32	4.73 ± 0.65	4.06 ± 0.98	3.66 ± 0.79
	Δ	1.68 ± 1.30^a^*^^	0.89 ± 0.88^b^*^^	0.41 ± 0.70^c^	−0.38 ± 0.60^d^*^^
EPA (%)	Baseline	0.58 ± 0.23	0.67 ± 0.29	0.60 ± 0.19	0.56 ± 0.14
	Week 16	0.74 ± 0.21	0.70 ± 0.36	0.54 ± 0.26	0.47 ± 0.24
	Δ	0.16 ± 0.26^a^*^^	0.06 ± 0.22^a^	−0.07 ± 0.23^b^	−0.09 ± 0.20^b^*^^
ALA (%)	Baseline	0.14 ± 0.13	0.15 ± 0.13	0.16 ± 0.14	0.14 ± 0.14
	Week 16	0.15 ± 0.14	0.18 ± 0.14	0.12 ± 0.14	0.13 ± 0.14
	Δ	0.00 ± 0.16^a^	0.02 ± 0.14^a^*^^	−0.04 ± 0.16^a^	−0.01 ± 0.12^a^
DPA (%)	Baseline	2.48 ± 0.41	2.40 ± 0.46	2.43 ± 0.50	2.35 ± 0.37
	Week 16	2.08 ± 0.30	2.04 ± 0.27	2.23 ± 0.38	2.16 ± 0.28
	Δ	−0.41 ± 0.40^a^*^^	−0.36 ± 0.35^a^*^^	−0.19 ± 0.35^b^*^^	−0.19 ± 0.30^b^*^^

*Values are means ± SD. Red blood cell ω3 profile parameter changes were analyzed using ANCOVA with treatment and smoking status as fixed effects, and the baseline parameter value as a covariate. P_baseline_ was <0.01 for all parameters, P_treatment_ was <0.01 for all parameters except ALA, and P_smoking_ was only <0.05 for ALA. Values without a common letter (a, b, c, d) indicate significant between-group differences (P < 0.05).*

* *Indicates significant within-group change from baseline to week 16 (P < 0.05). At baseline, n = 31, 30, 31, and 33 for high-dose, mid-dose, low-dose, and placebo groups; at week 16, n = 29, 30, 28, and 32 for high-dose, mid-dose, low-dose, and placebo groups, respectively. ALA, alpha-linolenic acid; DHA, docosahexaenoic acid; DPA, docosapentaenoic acid; EPA, eicosapentaenoic acid; Δ, change from baseline*.

### 3.6. No Clinically Significant Changes in Conventional Serum Cardiovascular Biomarkers After 4 and 16 Weeks of LC-ω3-Rich Canola Oil Supplementation

The concentrations of conventional serum cardiovascular biomarkers before and after 4 and 16 weeks of LC-ω3-rich canola oil supplementation are presented in Table 6. Changes in TG concentrations after 16 weeks of supplementation were not different between or within study groups (*P* > 0.05); no difference was observed in the between- or within-group comparisons in TG after 4 weeks of supplementation either. Regarding other serum cardiovascular biomarkers, the high-dose group exhibited an increase in total cholesterol (*P* = 0.0086), LDL-C (*P* = 0.0144), and LDL-C/HDL-C ratio (*P* = 0.0336) at week 4, but only the increase in total cholesterol remained at week 16 (*P* = 0.0103). The mid-dose group only showed an increase in hsCRP at week 4 (*P* = 0.0302) and no changes were observed in any cardiovascular biomarkers at week 16 (*P* > 0.05). The low-dose group showed no changes at week 4 (*P* > 0.05) but a reduction in HDL-C and an increase in the LDL-C/HDL-C ratio at week 16 (both *P* < 0.05). Lastly, the placebo group exhibited an increase in hsCRP at week 16 (*P* = 0.0025), without changes in any other cardiovascular biomarkers (*P* > 0.05). Between-group differences were observed for the overall changes from baseline in TC and HDL-C, but the results were inconclusive regarding the between-group comparisons at specific visits.

**Table 6:** Serum concentrations of cardiovascular biomarkers before and after 4- and 16-week supplementation of LC-ω3-rich canola oil or placebo.

	**Study visit**	**High-dose**	**Mid-dose**	**Low-dose**	**Placebo**
TG (mmol/L)	Baseline	1.18 ± 0.84	1.10 ± 0.64	1.04 ± 0.49	1.04 ± 0.44
	Week 4	1.16 ± 0.71	1.12 ± 0.54	1.06 ± 0.51	1.10 ± 0.50
	Week 16	1.29 ± 0.93	1.07 ± 0.42	1.04 ± 0.59	1.04 ± 0.54
	ΔWeek 4	−0.02 ± 0.45	0.02 ± 0.37	0.01 ± 0.25	0.06 ± 0.40
	ΔWeek 16	0.06 ± 0.38	−0.05 ± 0.56	−0.02 ± 0.32	−0.00 ± 0.38
TC (mmol/L)	Baseline	4.61 ± 1.02	4.86 ± 1.20	4.64 ± 1.02	4.54 ± 0.87
	Week 4	4.81 ± 1.02	4.91 ± 1.15	4.57 ± 0.99	4.48 ± 0.88
	Week 16	4.90 ± 1.14	4.74 ± 1.14	4.63 ± 1.15	4.45 ± 0.91
	ΔWeek 4	0.20 ± 0.46^*^	0.05 ± 0.39	−0.06 ± 0.44	−0.06 ± 0.46
	ΔWeek 16	0.20 ± 0.71^*^	−0.12 ± 0.64	−0.08 ± 0.51	−0.06 ± 0.57
LDL-C (mmol/L)	Baseline	2.50 ± 0.96	2.73 ± 1.04	2.63 ± 0.88	2.54 ± 0.72
	Week 4	2.66 ± 0.83	2.73 ± 0.99	2.59 ± 0.86	2.44 ± 0.71
	Week 16	2.66 ± 1.01	2.61 ± 1.00	2.69 ± 0.99	2.43 ± 0.71
	ΔWeek 4	0.16 ± 0.44^*^	0.01 ± 0.33	−0.04 ± 0.36	−0.09 ± 0.44
	ΔWeek 16	0.10 ± 0.68	−0.13 ± 0.51	0.02 ± 0.38	−0.09 ± 0.40
HDL-C (mmol/L)	Baseline	1.57 ± 0.50	1.64 ± 0.41	1.53 ± 0.37	1.53 ± 0.45
	Week 4	1.62 ± 0.51	1.67 ± 0.35	1.51 ± 0.33	1.54 ± 0.48
	Week 16	1.66 ± 0.58	1.65 ± 0.36	1.46 ± 0.32	1.55 ± 0.45
	ΔWeek 4	0.05 ± 0.14	0.03 ± 0.23	−0.03 ± 0.15	0.01 ± 0.17
	ΔWeek 16	0.07 ± 0.15	0.04 ± 0.24	−0.08 ± 0.18^*^	0.03 ± 0.24
LDL-C to HDL-C ratio	Baseline	1.72 ± 0.71	1.75 ± 0.69	1.79 ± 0.64	1.79 ± 0.71
	Week 4	1.80 ± 0.77	1.68 ± 0.58	1.79 ± 0.66	1.75 ± 0.82
	Week 16	1.74 ± 0.73	1.63 ± 0.61	1.90 ± 0.69	1.70 ± 0.78
	ΔWeek 4	0.08 ± 0.42^*^	−0.07 ± 0.40	−0.00 ± 0.31	−0.05 ± 0.32
	ΔWeek 16	−0.00 ± 0.48	−0.14 ± 0.54	0.10 ± 0.27^*^	−0.09 ± 0.29
hsCRP (mmol/L)	Baseline	1.76 ± 2.22	2.35 ± 5.56	1.46 ± 1.56	1.59 ± 2.40
	Week 4	1.73 ± 2.26	3.04 ± 9.38	1.45 ± 1.33	1.58 ± 1.69
	Week 16	1.49 ± 1.72	2.43 ± 5.37	1.80 ± 2.08	4.04 ± 13.94
	ΔWeek 4	−0.03 ± 1.39	0.68 ± 10.78^*^	−0.01 ± 1.22	−0.01 ± 1.38
	ΔWeek 16	−0.42 ± 1.34	0.02 ± 7.75	0.26 ± 1.64	2.44 ± 13.74^*^

*Values are means ± SD. The changes in serum cardiovascular biomarkers on the two different visits (4 and 16 weeks) were analyzed using an ANCOVA model with treatment, visit, smoking status, and interaction between treatment and visit (treatment × visit) as fixed effects, and the baseline parameter value as a covariate. P_treatment_, P_visit_, and P_treatment × visit_ were mostly >0.05 except P_treatment_ was <0.05 for TC and HDL-C, P_smoking_ was only <0.05 for LDL-C to HDL-C ratio and hsCRP, while P_baseline_ was <0.0001 for all parameters. No significant between-group difference was observed.*

* *Indicates significant within-group change from baseline to the study visit (P < 0.05). At baseline and week 4, n = 31, 30, 31, and 33 for high-dose, mid-dose, low-dose, and placebo groups; at week 16, n = 29, 30, 28, and 32 for high-dose, mid-dose, low-dose, and placebo groups, respectively. HDL-C, high density lipoprotein cholesterol; hsCRP, high-sensitivity C-reactive protein; LDL-C, low density lipoprotein cholesterol; TC, total cholesterol; TG, triglycerides; Δ, change from baseline*.

### 3.7. Sixteen-Week Daily Supplementation of LC-ω3-Rich Canola Oil Was Safe and Well-Tolerated

The LC-ω3-rich canola oil capsules were well tolerated; over 16 weeks of daily supplementation, most participants reported that the products were easy to swallow (92.9%), did not have an after taste (97.3%), did not cause belching or burping (92.7%), and did not cause heartburn or acid reflux (96.5%) (data not shown). One treatment-emergent adverse event (TEAE) (diarrhea, moderate in severity) in the mid-dose group was deemed related to test product by the blinded study investigator and resulted in participant withdrawal; the TEAE was resolved during the study. No TEAEs were deemed related in the high-dose, low-dose, or placebo group. No serious adverse events or deaths were reported. Changes in clinical laboratory evaluation, vital signs, and body measurements over the study period were comparable across the study groups (data not shown).

## 4. Discussion

Blood lipid and fatty acid profiles play critical roles in human health and they can be modulated by dietary changes, such as increasing LC-ω3 PUFA intake (1). Transgenic plants can serve as a viable and more sustainable source of LC-ω3 PUFA (27, 28). Using a seamless phase I/II study design, a LC-ω3-rich oil sourced from transgenic canola seeds was evaluated in three doses for its pharmacokinetics and safety, while exploring its effects on serum cardiovascular biomarkers. Overall, this LC-ω3-rich canola oil demonstrated good dose-related bioavailability of DHA and improved whole blood (short-term) and RBC (long-term) ω3 fatty acid profiles, with minimal impacts on cardiovascular biomarkers. The 16-week supplementation was found safe and well-tolerated. This study demonstrated the ability of this LC-ω3-rich canola oil to increase circulating LC-ω3 PUFA (i.e., omega-3 whole blood score and omega-3 index) to an extent that may support cardiovascular and cognitive health, after 4 weeks of daily supplementation. Overall, this study has demonstrated that LC-ω3-rich canola oil increases ω3 nutritional status and is a good candidate to address the ω3 nutrient intake gap in adults.

A linear response to the different doses of LC-ω3-rich canola oil supplementation was seen for DHA AUC_0−72*h*_ and EPA AUC_0−72*h*_, which is in line with the PK profile characteristics observed when LC-ω3 PUFA are delivered from fish oil sources (29). Shorter term ω3 absorption was further evidenced by an increase in the concentrations of whole blood DHA, EPA, omega-3 whole blood score, and omega-3 index after 4 weeks of high-dose and mid-dose supplementation. These results are corroborated by another recent clinical study showing good bioavailability of DHA and EPA from an ω3-rich transgenic Camelina sativa seed oil in healthy adults over 8 weeks (14). Furthermore, longer term ω3 absorption after a full RBC turn-over was evidenced by an increase in RBC proportions of DHA, EPA and ALA following 16 weeks of supplementation, which was similar to other ω3 supplementation studies (30–32). The decrease of RBC DPA in high- and mid-dose groups is in agreement with the observation in another study, which showed that 10-week supplementation with high-dose DHA increased the omega-3 index but reduced DPA, as compared to high-dose EPA supplementation or placebo (corn oil) (32). Overall, these results show that this LC-ω3-rich canola oil is a viable source of bioavailable DHA and EPA, and these responses were analogous to marine-sourced TG ω3 formulations.

Modification of the omega-3 index, and omega-3 whole blood score are correlated with positive health outcomes. Increases in omega-3 whole blood score have been associated with decreased risk of sudden cardiac death in men with no history of cardiovascular events (33). Similarly, increases in omega-3 index have been associated with decreased risks of CVD, mortality (any-cause) (34), and age-related cognitive decline (35). Based on the omega-3 index risk category reported previously for cardiovascular-related issues (34, 36), the omega-3 index of the high-dose group in the present study moved from the “moderate risk” category pre-supplementation to the “low risk” category post-supplementation. For cognitive deterioration with aging, the omega-3 index of the high-dose and mid-dose groups moved from the “high risk” to the “low risk” category as well (35). Future studies on the potential effects of this LC-ω3-rich canola oil on these health risks may be warranted.

The co-consumption of LC-ω3 PUFA and canola oil has been shown to improve lipid profiles in a population with CVD risks (37), which was not observed in the current study. This is consistent with the lack of changes in lipid profiles observed in another study, in which healthy adults showed increased omega-3 index after ~5 months of DHA+EPA supplementation (30). A probable explanation for this is the generally healthy status of participants in this study, where cardiovascular biomarkers were not assessed as an inclusion/exclusion criterion given that the focus of the study was to examine this LC-ω3 canola oil in healthy adults. Future studies in populations with CVD risks are warranted to elicit the effects of this LC-ω3-rich canola oil on cardiovascular biomarkers.

The global demand for LC-ω3 PUFA, which exceeds 1.27 million tones per year, cannot be met by current ocean-based supplies (12). Notably, the highest dose of LC-ω3-rich canola oil used in the present study provided 380 mg LC-ω3 PUFA (360 mg DHA + 20 mg EPA), thereby highlighting its potential to help fill the supply-demand gap. Marine-based LC-ω3 PUFA in the supplement industry are mainly sourced from small fishes, but their overfishing can reduce the food source for larger marine animals and subsequently compromise the supply chain (38). The significant shortage for LC-ω3 PUFA supply and the damage to the ocean habitat can be addressed by utilizing sustainable and scalable sources of LC-ω3 PUFA, such as the transgenic LC-ω3-rich canola oil. It is notable that this oil was well-tolerated; common complaints with fish oil supplementation include fishy after taste, burps, acid reflux, and gastrointestinal upset (39), but these symptoms were not experienced with the LC-ω3 canola oil capsules in the present study, highlighting their potential consumer acceptability.

There are limitations to the study. Given the inclusion of generally healthy participants in this study, it was difficult to observe effects on cardiovascular biomarkers. Nonetheless, this is the first human study with this novel transgenic canola oil, hence the data in healthy individuals were needed to establish its pharmacokinetics and safety. Another potential weakness of this study is the use of corn oil as placebo. While conventional canola oil could have been used as a comparator for the transgenic canola oil containing LC-ω3 PUFA, corn oil was chosen for its lack of ω3 fatty acids. For future studies, it would be beneficial to compare this plant-based, LC-ω3-rich oil with fish oil or other traditional, marine-based sources of LC-ω3 PUFA as well.

## 5. Conclusions

The present findings in both acute pharmacokinetics and longer-term efficacy show that this LC-ω3-rich oil from transgenic canola seeds improved blood ω3 profiles within 72 h after a single dose, and after 4 and 16 weeks of daily supplementation. While clinically relevant changes in cardiovascular biomarkers in this healthy adult population were not observed, significant improvements in ω3 nutrition status were demonstrated. Given its sustainable and scalable production, good safety profile and consumer acceptability, and the ability to improve blood ω3 profile in the generally healthy population presented herein, this LC-ω3-rich canola oil is a highly promising source to help fill the ω3 nutrient gap in the U.S. and globally.

## Data Availability Statement

The original contributions presented in the study are included in the article/Supplementary Material, further inquiries can be directed to the corresponding author/s.

## Ethics Statement

The studies involving human participants were reviewed and approved by Canadian Shield Ethics Review Board (OHRP Registration IORG0003491, Burlington, Ontario). The participants provided their written informed consent to participate in this study.

## Author Contributions

JB: conceptualization and methodology. AB: investigation. JB and BH: data analysis and curation. XL and DV: writing—original draft preparation. XL, JB, AB, DV, and BH: writing—review and editing. JB and AB: supervision. All authors have read and agreed to the published version of the manuscript. All authors agree to be accountable for the content of the work.

## Funding

The study was funded by Nuseed^®^.

## Conflict of Interest

JB, AB, XL, and DV are employees of Nutrasource Pharmaceutical and Nutraceutical Services Inc., a contract research organization. BH is a consultant for Nuseed^Ⓡ^ and declares no other conflicts of interest relevant to this article. Nuseed^Ⓡ^ reviewed and commented on the study design but did not take part in the study implementation, or in the collection, analysis, and interpretation of study data.

## Publisher's Note

All claims expressed in this article are solely those of the authors and do not necessarily represent those of their affiliated organizations, or those of the publisher, the editors and the reviewers. Any product that may be evaluated in this article, or claim that may be made by its manufacturer, is not guaranteed or endorsed by the publisher.
