# Supplementary material for: Transgenic Canola Oil Improved Blood Omega-3 Profiles: A Randomized, Placebo-Controlled Trial in Healthy Adults
Source: Front Nutr. 2022 Mar 10;9:847114. doi: 10.3389/fnut.2022.847114 (PMC8960439; doi:10.3389/fnut.2022.847114)
Supplement: Supplementary file 1 [file Table_1.docx]

Supplementary table

Table 1. Dose linearity of test products over a 72-hour post-dose PK period^1^

|  |  |  | Logarithm-transformed values | | | Between-group comparison |
| --- | --- | --- | --- | --- | --- | --- |
|  |  |  | High-dose | Mid-dose | Low-dose | P-value |
| DHA | AUC_0-72h_ (ug*hr/mL) | N | 32 | 31 | 30 | 0.0011 |
|  |  | Mean ± SD | 5.67 ± 0.763 | 4.96 ± 1.543 | 4.63 ± 1.230 |  |
|  |  | 95% CI | 5.39, 5.94 | 4.40, 5.53 | 4.17, 5.09 |  |
|  | C_max_ (ug/mL) | N | 32 | 31 | 30 | <.0001 |
|  |  | Mean ± SD | 2.90 ± 0.648 | 2.42 ± 0.665 | 2.07 ± 0.583 |  |
|  |  | 95% CI | 2.67, 3.14 | 2.17, 2.66 | 1.86, 2.29 |  |
| EPA | AUC_0-72h_ (ug*hr/mL) | N | 30 | 28 | 26 | 0.0092 |
|  |  | Mean ± SD | 3.80 ± 1.256 | 3.07 ± 1.973 | 2.43 ± 2.487 |  |
|  |  | 95% CI | 3.33, 4.27 | 2.30, 3.83 | 1.42, 3.43 |  |
|  | C_max_ (ug/mL) | N | 30 | 28 | 26 | 0.1895 |
|  |  | Mean ± SD | 0.99 ± 0.757 | 0.63 ± 1.288 | 0.58 ± 1.413 |  |
|  |  | 95% CI | 0.70, 1.27 | 0.14, 1.13 | 0.01, 1.15 |  |

^1^Values are means ± SD of logarithm-transformed AUC_0-72h_ and C_max._ Linear regressions were performed with log_[AUC0-72h]_ and log_[Cmax]_ as dependent variables with log_(dose)_ as the independent variable. *P*<0.05 indicates a significant linear relationship between log_(dose)_ and the logarithm-transformed PK parameters. AUC, area under the curve; C_max_, peak concentration; DHA, docosahexaenoic acid; EPA, eicosapentaenoic acid; PK, pharmacokinetics.
