# Supplementary material for: Transgenic Canola Oil Improved Blood Omega-3 Profiles: A Randomized, Placebo-Controlled Trial in Healthy Adults
Source: Front Nutr. 2022 Mar 10;9:847114. doi: 10.3389/fnut.2022.847114 (PMC8960439; doi:10.3389/fnut.2022.847114)
Supplement: Supplementary file 2 [file Table_2.docx]

Supplementary table

Table 2. Dose proportionality of test products over a 72-hour post-dose PK period^1^

|  |  |  | Dose-adjusted and logarithm-transformed values | | | Between-group comparison |
| --- | --- | --- | --- | --- | --- | --- |
|  |  |  | High-dose | Mid-dose | Low-dose | P-value |
| DHA | AUC_0-72h_ (ug*hr/mL) | N | 32 | 31 | 30 | 0.4302 |
|  |  | Mean ± SD | 5.67 ± 0.763 | 5.66 ± 1.543 | 6.01 ± 1.230 |  |
|  |  | 95% CI | 5.39, 5.94 | 5.09, 6.22 | 5.55, 6.47 |  |
|  | C_max_ (ug/mL) | N | 32 | 31 | 30 | 0.0034 |
|  |  | Mean ± SD | 2.90 ± 0.648 | 3.11 ± 0.665 | 3.46 ± 0.583 |  |
|  |  | 95% CI | 2.67, 3.14 | 2.87, 3.36 | 3.24, 3.68 |  |
| EPA | AUC_0-72h_ (ug*hr/mL) | N | 30 | 28 | 26 | 0.9947 |
|  |  | Mean ± SD | 3.80 ± 1.256 | 3.76 ± 1.973 | 3.81 ± 2.48 |  |
|  |  | 95% CI | 3.33, 4.27 | 3.00, 4.53 | 2.81, 4.82 |  |
|  | C_max_ (ug/mL) | N | 30 | 28 | 26 | 0.0093 |
|  |  | Mean ± SD | 0.99 ± 0.757 | 1.33 ± 1.288 | 1.97 ± 1.413 |  |
|  |  | 95% CI | 0.70, 1.27 | 0.83, 1.83 | 1.39, 2.54 |  |

^1^Values are means ± SD of logarithm-transformed and dose-adjusted AUC_0-72h_ and C_max._ Before the logarithm-transformation, the original AUC_0-72h_ and C_max_ values were adjusted using dose-adjustments in the following manner: high-dose (four capsule of test product): dose adjusted AUC_0-72h_ (or C_max_) = 1 × AUC_0-72h_ (or C_max_); mid dose (two capsule of test product): dose adjusted AUC_0-72h_ (or C_max_) = 2 × AUC_0-72h_ (or C_max_); low dose (one capsule of test product): dose adjusted AUC_0-72h_ (or C_max_) = 4 × AUC_0-72h_ (or C_max_). ANOVA was used to compare the logarithm-transformed, dose-adjusted values between different test product groups. *P*<0.05 indicates significant difference between groups (i.e., lack of dose proportionality). AUC, area under the curve; C_max_, peak concentration; DHA, docosahexaenoic acid; EPA, eicosapentaenoic acid; PK, pharmacokinetics.
